# Heart Donation After Controlled Circulatory Death in Noncardiac Surgery Hospitals With a Mobile Heart Team: A Case Series From an Italian Regional Program and Integrated Review of the Literature

**DOI:** 10.1111/aor.70255

**Published:** 2026-09-27

**Authors:** Maria Benedetto, Valentina Roncarati, Arianna Campana, Erika Cordella, Domenico Nocera, Sofia Martin‐Suarez, Luca Botta, Davide Pacini, Letizia Perinati, Massimo Baiocchi, Tommaso Tonetti, Fabrizio Monaco

**Affiliations:** ^1^ Cardio‐Thoracic and Vascular Anesthesia and Intensive Care Unit IRCCS Azienda Ospedaliero‐Universitaria di Bologna Bologna Italy; ^2^ Coordination for Transplants Center (CRT‐ER) IRCCS Azienda Ospedaliero‐Universitaria di Bologna Bologna Italy; ^3^ Anesthesiology and General Intensive Care Unit IRCCS Azienda Ospedaliero‐Universitaria di Bologna Bologna Italy; ^4^ Division of Cardiac Surgery, Cardiac Surgery Department IRCCS Azienda Ospedaliero Universitaria di Bologna Bologna Italy; ^5^ Department of Medical and Surgical Sciences Alma Mater Studiorum University of Bologna Bologna Italy; ^6^ Division of Cardiology, Department of Medicine Verona University Hospital Verona Italy; ^7^ Anesthesiology and General Intensive Care Unit Ospedale Infermi di Rimini, AUSL della Romagna Rimini Italy

**Keywords:** circulatory arrest, heart transplant, organ donation, regional perfusion

## Abstract

Controlled donation after circulatory death (cDCD) with thoracoabdominal normothermic regional perfusion (TA‐NRP) has emerged worldwide as a strategy to expand the heart donor pool. In Italy, however, its implementation is constrained by a particularly restrictive setting, shaped by the mandatory 20‐min asystolic no‐touch period and by the absence of cardiac surgery units in most donor hospitals. Against this backdrop, the Emilia‐Romagna Regional Transplant Network developed a mobile DCD Heart Team capable of performing TA‐NRP in peripheral hospitals. We report a case series of 19 cDCD donors managed with TA‐NRP within a regional program based at a high‐volume tertiary university hospital, all with prolonged functional warm ischemic time (FWIT). Seventeen hearts (89.5%) were successfully procured and transplanted, with a median FWIT of 41 (37.5–52) minutes, and serial transesophageal echocardiography and hemodynamic assessment supported real‐time monitoring of myocardial recovery. No recipient required postcardiotomy veno‐arterial extracorporeal membrane oxygenation (VA‐ECMO), and no primary graft dysfunction or 30‐day mortality were observed. These findings support TA‐NRP heart procurement as a feasible and clinically effective procedure—even in centers without onsite cardiac surgery—when a dedicated mobile heart team is available, thereby encouraging the broad adoption of cDCD heart procurement within the Italian legislative and organizational context.

## Graphical abstract

In this series of 19 DCD donors using TA‐NRP, 89.5% of hearts were successfully transplanted with no PGD, no postoperative ECMO, and no 30‐day mortality, demonstrating the feasibility and safety of this approach.

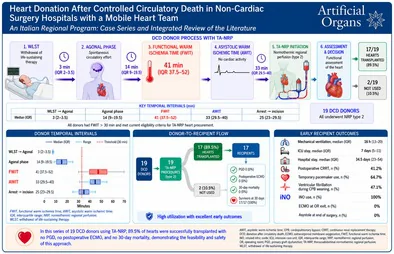

## 1 Introduction

In the context of heart transplantation, limited by the shortage of suitable donors, controlled donation after circulatory death (cDCD) has emerged as a strategy to expand the donor pool. Early experiences with DCD heart transplantation, using either ex situ heart perfusion (ESHP) or thoracoabdominal normothermic regional perfusion (TA‐NRP), have shown that donor hearts can recover satisfactory function after circulatory arrest [1, 2]. By restoring coronary perfusion while excluding cerebral circulation, TA‐NRP dynamically assesses myocardial recovery, hemodynamic response, and correction of metabolic derangements. In established national programs in the United Kingdom, Spain, Australia, and, more recently, the United States, outcomes after cDCD heart transplantation have approached those reported after donation after brain death (DBD) [3, 4], and direct evaluation of the heart under physiologic loading conditions has also enhanced clinician confidence in accepting cDCD donor hearts. In Italy, however, the implementation of cDCD heart transplantation faces important challenges. One major issue is the legal framework for death determination, certified on the basis of irreversible cessation of all brain functions. This translates into a mandatory 20‐min no‐touch period—longer than that used in most other reported programs—of continuous electrocardiographic asystole before death can be declared [5, 6, 7], inevitably prolonging functional warm ischemia time (FWIT) and potentially compromising myocardial viability. Furthermore, the structural organization of the Italian healthcare system expects intensive care units (ICUs) to be located also in hospitals without onsite cardiac surgery, lacking the equipment and expertise to perform such procedure independently. Whereas DBD donors may be transferred to a transplant center with circulation preserved, cDCD heart procurement with TA‐NRP must be initiated immediately after death certification in the donor hospital. This requires extracorporeal circulation, endovascular or surgical exclusion of cerebral blood flow, median sternotomy, and advanced echocardiographic evaluation. The pathway is made even more complex by the procedural requirements surrounding withdrawal of life‐sustaining treatment (WLST). International best practices require clear separation between the team responsible for WLST and death determination, and the team involved in retrieval and transplantation. In parallel, palliative care must be delivered with rigor and transparency in order to preserve dignity at the end of life and maintain public trust [8, 9, 10]. Coordinating these processes becomes demanding when multiple mobile teams operate across different hospitals. To address these challenges, the Emilia‐Romagna Regional Transplant Network developed a mobile DCD Heart Team, composed of two cardiac surgeons, a cardiac anesthesiologist, a perfusionist, and a cardiac scrub nurse, able to travel to donor hospitals without onsite cardiac surgery and perform TA‐NRP locally. This model mirrors successful experiences in Australia and parts of the United Kingdom, where centralized expertise supports procurement across a regional network. In such programs, the anesthesiologist has a central role, linking donor critical care, extracorporeal membrane oxygenation (ECMO) support, intraoperative hemodynamic management, and transesophageal echocardiographic assessment [11, 12]. In Italy, current literature on successful TA‐NRP heart procurement performed by a mobile team in hospitals without onsite cardiac surgery is scarce. In this study, we report our experience with 19 cDCD donors managed with TA‐NRP within a regional program that included hospitals without onsite cardiac surgery and was supported by a mobile heart team. We describe the organizational pathway, donor management, ischemic intervals, reperfusion course, and early posttransplant outcomes, discussing these findings in the context of the international experience available.

## 2 Materials and Methods

### 2.1 Program Structure and Operational Workflow

The program was developed within the Emilia‐Romagna Regional Transplant Network as a hub‐and‐spoke model, with the Bologna Heart Transplant Center serving as the hub and peripheral ICUs—without onsite cardiac surgery—serving as procurement sites. A mobile DCD Heart Team operated according to a structured activation pathway, consisting of notification by the regional transplant coordination system, immediate mobilization, arrival at the donor hospital before WLST, coordination with local ICU clinicians in charge of WLST and death certification, and setup for femoral cannulation, VA‐ECMO initiation, cerebral exclusion, and TA‐NRP. This pathway was designed to minimize delays while preserving the mandatory separation between WLST and procurement teams. The study was approved by the Ethics Committee of Area Vasta Emilia Centro (AVEC) with protocol number 88/2026/AOUBo. In accordance with Italian transplantation law, all donors had either provided documented consent during their lifetime or, in the absence of explicit consent, non‐opposition was ascertained from legally entitled relatives.

### 2.2 Donor Selection

Eligible donors were Maastricht Category III (controlled DCD). Inclusion criteria were age < 65 years although not mandatory in selected cases, left‐ventricular ejection fraction (LVEF) ≥ 50%, no history of ischemic cardiomyopathy, significant valvular disease, or previous cardiac surgery, and no contraindications to organ donation (e.g., malignancy).

Evaluation consisted of ECG, transthoracic or transesophageal echocardiography, and review of comorbidities. Additional testing, such as coronary CT angiography or invasive coronary angiography, was performed when indicated. Consent was obtained as per national legislation.

### 2.3 Withdrawal of Life‐Sustaining Treatment Procedure

WLST was performed exclusively by the local ICU team. Palliative sedation, consisting of propofol or midazolam combined with opioids, was achieved in accordance with SIAARTI (Italian Society of Anesthesia, Intensive Care and Pain Management) guidelines [13]. Routine monitoring including invasive arterial pressure, ECG, and pulse oximetry was established and maintained by the local ICU team. After arrival, the visiting cardiac anesthesiologist inserted the pulmonary artery catheter and the transesophageal echocardiography (TEE) probe before WLST to enable real‐time assessment of cardiac activity. After circulatory arrest, the legally mandated 20‐min no‐touch time was strictly observed. Death was certified by ICU physicians not involved in procurement.

### 2.4 Definitions

According to hospital practice, total warm ischemia time (tWIT) was defined as the time from withdrawal of life support to the initiation of NRP, whereas FWIT was defined as the interval from the onset of the agonal phase to the initiation of NRP. The agonal phase was defined as the period beginning with the development of hypotension—systolic arterial pressure below 50 mmHg—or desaturation—peripheral oxygen saturation below 75%—whichever occurred first. The circulatory arrest‐to‐surgical incision interval was defined as the time from the onset of asystole to the first surgical incision and included the mandatory 20‐min no‐touch period. Asystolic warm ischemic time (AWIT) was defined as the time from the onset of asystole to the initiation of NRP. Thirty‐day mortality was defined as death from any cause occurring within 30 days after transplantation. Severe PGD and vasoactive‐inotropic score (VIS) were defined according to the International Society of Heart and Lung Transplantation criteria [14].

### 2.5 TA‐NRP Procedure

After death certification, femoral cannulation for VA‐ECMO was performed and surgical exclusion of the supra‐aortic vessels was performed according to the institutional protocol. An endovascular aortic occlusion balloon was used to prevent cerebral reperfusion until surgical clamping of the brachiocephalic trunk, left common carotid artery, and left subclavian artery was completed. VA‐ECMO was subsequently initiated to establish TA‐NRP. The NRP circuit was adapted to a mobile closed extracorporeal configuration, allowing the procedure to be performed in peripheral hospitals [15].

The same standardized reperfusion protocol was applied to all donors. At TA‐NRP initiation, extracorporeal blood flow was promptly increased to approximately 80% of the estimated cardiac output calculated according to donor body surface area, accounting for exclusion of the cerebral circulation. Post‐oxygenator blood temperature was targeted at 37°C. Fresh‐gas flow and oxygen concentration were adjusted according to serial blood‐gas analyses to restore normocapnia and maintain adequate oxygen delivery. A 20‐Fr left‐ventricular vent was inserted through the right superior pulmonary vein and connected to the venous drainage cannula to provide ventricular unloading during early reperfusion. As myocardial contractility recovered, extracorporeal support was progressively reduced. Relative to the initial extracorporeal flow, nominal flow targets were approximately 80% at 30 min, 45% at 60 min, and 35% at 90 min. Actual flow was adjusted according to donor body size, venous return, hemodynamic conditions, and serial TEE findings. Pump‐speed reduction and partial clamping of the venting line allowed gradual biventricular reloading. Metabolic recovery was assessed through serial measurements of lactate, pH, base excess, and blood gases. Acid–base disturbances and abnormalities in electrolytes, temperature, ventilation, intravascular volume, hemoglobin, and cardiac rhythm were corrected as clinically indicated. Vasoactive and inotropic support was individualized according to hemodynamic and echocardiographic findings. Electrical defibrillation or temporary pacing was used when required. No dedicated pharmacological post‐conditioning protocol was applied. After satisfactory myocardial recovery, TA‐NRP was progressively weaned and discontinued. The extracorporeal circuit remained functional through recirculation across the arteriovenous bridge, allowing support to be resumed if necessary. Cardiac function was subsequently assessed under native biventricular loading conditions for approximately 45–60 min before the final decision regarding graft acceptance and procurement.

### 2.6 Role of the Cardiac Anesthesiologist

The cardiac anesthesiologist coordinated invasive hemodynamic monitoring, mechanical ventilation, correction of acid–base and electrolyte abnormalities, fluid and temperature management, rhythm stabilization, and vasoactive or inotropic support during TA‐NRP. Serial TEE examinations evaluated biventricular systolic function, regional wall motion, interventricular septal dynamics, ventricular distension, and valvular competence during reperfusion and progressive ECMO weaning. This role provided continuity between donor critical care, extracorporeal reperfusion, and functional graft assessment, particularly at procurement sites without onsite cardiac surgery. The timing and criteria used for myocardial assessment are described in the following section and Table 1.

**TABLE 1:** Multiparametric criteria for myocardial recovery and graft acceptance during TA‐NRP.

Domain	Parameter	Reference target supporting acceptance	Finding arguing against acceptance
Recovery period	Maximum TA‐NRP duration	Functional recovery within ≤ 180 min	Failure to recover within 180 min
Native‐loading assessment	Observation after ECMO weaning	Stable function for approximately 45–60 min	Functional deterioration after support reduction or withdrawal
LV systolic function	LVEF	≥ 50%	Persistent LVEF < 50%
Regional LV function	Regional wall motion	Recovery of regional contractility without a clinically significant persistent abnormality	Persistent major regional wall‐motion abnormality
RV global function	RV size and systolic function	Normal or recovering RV size and systolic function	Persistent severe RV dilation or systolic dysfunction
RV fractional area change	FAC	≥ 40%	Persistent FAC < 40%
RV longitudinal function	TAPSE	Interpreted serially; > 16 mm according to loading conditions	Persistent deterioration together with other evidence of RV dysfunction
Septal dynamics	Septal position and motion	Physiological septal behavior after biventricular reloading	Persistent abnormal septal displacement indicating ventricular imbalance
Valvular function	Valve morphology and competence	Absence of severe valvular disease	Severe structural or functional valvular dysfunction
Systemic pressure	MAP	≥ 60 mmHg under native loading conditions	Persistent MAP < 60 mmHg despite optimization
Cardiac performance	Cardiac index	≥ 2.5 L/min/m^2^ after ECMO weaning	Persistent cardiac index < 2.5 L/min/m^2^
Right‐sided filling pressure	CVP	< 12 mmHg	Persistent CVP ≥ 12 mmHg despite optimization
Left‐sided filling pressure	PAWP	< 12 mmHg	Persistent PAWP ≥ 12 mmHg despite optimization
Metabolic recovery	Lactate	Stable or decreasing trend	Persistent increase despite restoration of perfusion and metabolic optimization
Acid–base recovery	pH and base excess	Progressive correction toward the physiological range	Persistent severe acidosis despite reperfusion
Pharmacological support	Vasoactive and inotropic requirements	Stable or decreasing support	Escalating support required to maintain the reference targets
Cardiac rhythm	Rhythm assessment	Stable organized rhythm, spontaneous or supported	Persistent refractory arrhythmia
Final decision	Multidisciplinary evaluation	Concordant echocardiographic, hemodynamic, and metabolic recovery	Persistent clinically significant abnormality in one or more decisive domains

*Note:* Numerical parameters were used as reference targets within an integrated assessment and not as isolated absolute criteria.

Abbreviations: FAC, fractional area change; LVEF, left‐ventricular ejection fraction; MAP, mean arterial pressure; PAWP, pulmonary artery wedge pressure; TA‐NRP, thoracoabdominal normothermic regional perfusion; TAPSE, tricuspid annular plane systolic excursion.

### 2.7 Assessment of Myocardial Recovery and Graft Acceptance

Myocardial recovery was assessed serially during TA‐NRP, during progressive reduction of extracorporeal support, and after complete ECMO weaning. The final evaluation was performed under native biventricular loading conditions after approximately 45–60 min of observation. TA‐NRP could be maintained for up to 180 min when additional recovery time was required.

Graft acceptance was based on an integrated assessment of echocardiographic, hemodynamic, metabolic, and pharmacological‐support parameters (Table 1). TEE assessment included LVEF, global and regional left‐ventricular contractility, right‐ventricular size and systolic function, TAPSE, right‐ventricular fractional area change, interventricular septal dynamics, and valvular competence. Following ECMO weaning, the hemodynamic reference targets were MAP ≥ 60 mmHg, cardiac index ≥ 2.5 L/min/m^2^, CVP < 12 mmHg, and PAWP < 12 mmHg. Serial lactate measurements were interpreted as trends rather than according to a fixed clearance percentage or absolute threshold. Because lactate during TA‐NRP reflects whole‐body production and clearance, an isolated elevated value did not preclude graft acceptance when ventricular function, systemic perfusion, and acid–base status showed concordant recovery. Vasoactive and inotropic dose trends were also considered, with stable or decreasing support favoring acceptance and escalating requirements arguing against satisfactory recovery. Because this was a retrospective analysis of clinical practice, the numerical values were considered reference targets within a multidisciplinary assessment rather than isolated absolute criteria. No single echocardiographic, hemodynamic, or metabolic measurement independently determined graft acceptance or decline.

### 2.8 Collected Variables and Outcome Measures

Collected variables included demographics and clinical characteristics; recipient demographics, indication for transplantation, allocation and clinical status at transplantation, and preoperative mechanical circulatory support; donor–recipient sex, ABO group, Rhesus D antigen (RhD) status, age, weight, and predicted heart mass matching; procedural and ischemic intervals; procurement distance and travel time; and reperfusion‐related hemodynamic and TEE findings, donor VIS at separation from TA‐NRP support; and recipient VIS at operating room exit. Postoperative recipient outcomes included duration of mechanical ventilation, tracheostomy, ICU and hospital stay, primary graft dysfunction, renal replacement therapy, acute cellular rejection (ACR) at first biopsy and 30‐day mortality.

### 2.9 Statistical Analysis

All statistical analyses were conducted using R (R Foundation for Statistical Computing, Vienna, Austria). No formal hypothesis testing or comparative statistical analyses were performed, given the descriptive nature of the study and the limited sample size. All continuous variables are reported as median and IQR. Categorical variables were presented as counts and percentages.

## 3 Results

### 3.1 Donor and Procedural Characteristics

Baseline donor, recipient and donor–recipient matching characteristics are summarized in Table 2, with corresponding case‐level data provided as Tables S1A, S1B, and S1C. The 19 donors had a median age of 57 (IQR, 50–59.5) years, and 15 (78.9%) were male. The causes of death were postanoxic encephalopathy in 10 donors (52.6%) and cerebral hemorrhage in 9 (47.4%). Four donors (21.1%) had hypertension, and the median ICU stay before procurement was 11 days (IQR, 8–15). The median age of the 17 recipients was 57 years (IQR, 55–59), and 12 (70.6%) were male. The principal indications for transplantation were cardiomyopathy in seven recipients (41.2%) and coronary artery disease in 6 (35.3%). One recipient (5.9%) underwent transplantation under macro‐area urgent allocation. At transplantation, 14 recipients (82.4%) were outpatients, 2 (11.8%) were hospitalized on a regular ward, and 1 (5.9%) was in the ICU. None required preoperative temporary mechanical circulatory support. Fifteen donor–recipient pairs (88.2%) were sex matched, and all were ABO identical. RhD status was identical in 15 of 17 donor–recipient pairs (88.2%). The remaining two pairs (11.8%) involved an RhD‐negative donor and an RhD‐positive recipient. The median donor‐to‐recipient weight ratio was 1.07 (IQR, 1.01–1.10), and the median predicted heart mass ratio was 1.03 (IQR, 1.00–1.09). No graft had predicted heart mass undersizing greater than 10%.

**TABLE 2:** Baseline donor, recipient, and donor–recipient matching characteristics.

Characteristic	Value
**Donor characteristics**	***n* = 19**
Age, years	57 (50–59.5)
Male, *n* (%)	15 (78.9)
Weight, kg	82 (72–91.5)
Height, cm	175 (170–180)
Postanoxic encephalopathy, *n* (%)	10 (52.6)
Cerebral hemorrhage, *n* (%)	9 (47.4)
Hypertension, *n* (%)	4 (21.1)
ICU stay before procurement, days	11 (8–15)
**Recipient characteristics**	***n* = 17**
Age, years	57 (55–59)
Male sex, *n* (%)	12 (70.6)
**Indication for transplantation**	
Cardiomyopathy, *n* (%)	7 (41.2)
Coronary artery disease, *n* (%)	6 (35.3)
Valvular heart disease, *n* (%)	2 (11.8)
Congenital heart disease, *n* (%)	1 (5.9)
Other cardiovascular disease, *n* (%)	1 (5.9)
**Allocation status**	
Macro‐area urgent status, *n* (%)	1 (5.9)
Nonurgent status, *n* (%)	16 (94.1)
**Clinical setting at transplantation**	
Outpatient, *n* (%)	14 (82.4)
Hospitalized on a regular ward, *n* (%)	2 (11.8)
Hospitalized in ICU, *n* (%)	1 (5.9)
Preoperative MCS, *n* (%)	0 (0)
**Donor–recipient matching characteristics**	***n* = 17**
Same‐sex pairing, *n* (%)	15 (88.2)
Male donor–female recipient, *n* (%)	2 (11.8)
Female donor–male recipient, *n* (%)	0 (0)
Absolute age difference, years	8 (5–13)
Donor older than recipient, *n* (%)	9 (52.9)
ABO‐identical pairing, *n* (%)	17 (100)
ABO‐compatible nonidentical pairing, *n* (%)	0 (0)
ABO‐incompatible pairing, *n* (%)	0 (0)
Rh‐identical, *n* (%)	15 (88.2)
Rh‐nonidentical, *n* (%)	2 (11.8)
Donor‐to‐recipient weight ratio	1.07 (1.01–1.10)
Donor‐to‐recipient pHM ratio	1.03 (1.00–1.09)
pHM undersizing > 10%, *n* (%)	0 (0)

*Note:* Data are presented as median (interquartile range [IQR]) or *n* (%), as indicated.

Abbreviations: ABO, ABO blood group system; ICU, intensive care unit; MCS, mechanical circulatory support; pHM, predicted heart mass; Rh, Rhesus D antigen.

### 3.2 Graft Utilization and Reasons for Non‐Transplantation

All 19 donors included in the DCD cohort underwent normothermic regional perfusion (NRP type 2, *n* = 19). At separation from TA‐NRP support, the median donor VIS was 30 (IQR, 20–55). Of these, 17 hearts (89.5%) were successfully procured and transplanted, whereas two hearts (10.5%) were deemed unsuitable for transplantation. In one case, myocardial function recovered satisfactorily, but an unexpected thyroid malignancy identified during procurement was considered to confer an unacceptable transmission risk. This exclusion was unrelated to cardiac recovery. In the second case, contractile activity recovered, but TEE assessment under native loading conditions demonstrated severe mitral regurgitation; the graft was therefore declined for a cardiac reason.

### 3.3 Procedural, Ischemic, and Transport Characteristics

The main time intervals involved in DCD procurement are summarized in Table 3 (Figure 1), with corresponding case‐level data provided as Tables S2A, S2B, and S2C. The FWIT exceeded 30 min in all cases (100%), with a median duration of 41 min (IQR, 37.5–52). The median interval between WLST and the onset of the agonal phase was 3 min (IQR, 2–3.5), while the agonal phase lasted 14 min (IQR, 9–19.5). No donor exhibited a FWIT lower than 30 min, and all cases met current eligibility criteria for TA‐NRP heart procurement. The AWIT had a median duration of 33 min (IQR, 29.5–40). The median interval from the circulatory arrest to surgical incision was 25 min (IQR, 23–29.5). This interval included the mandatory 20‐min no‐touch period. Among the 17 transplanted hearts, median cold ischemic time was 189 min (IQR, 147–206). The donor hospitals were located a median of 83 km from the Bologna hub (IQR, 54–102), with a median estimated travel time of 64 min (IQR, 60–85; Figure 2).

**FIGURE 1:**
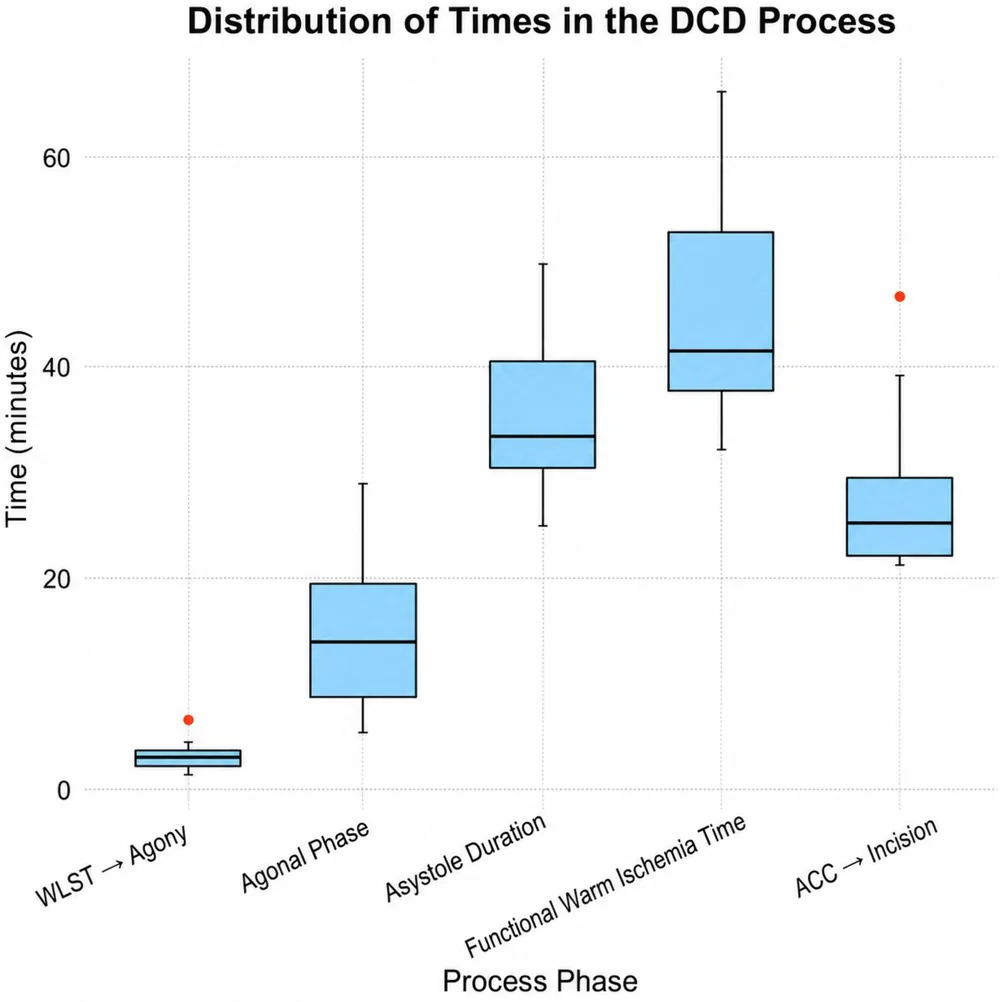
Distribution of time intervals during the controlled DCD process.

**TABLE 3:** Procedural, ischemic, transport, and early posttransplant outcomes in the cDCD heart transplantation program.

	Value
**Donor and procedural characteristics (*n* = 19)**	
Successfully transplanted hearts, *n* (%)	17 (89.5)
FWIT > 30 min, *n* (%)	19 (100)
FWIT, min	41 (37.5–52)
WLST to agonal phase, min	3 (2–3.5)
Agonal phase duration, min	14 (9–19.5)
Asystolic warm ischemic time (AWIT), min	33 (29.5–40)
Circulatory arrest to incision, min	25 (23–29.5)
Cold ischemic time, min	189 (147–206)
Donor VIS at separation from TA‐NRP support	30 (20–55)
Donor hospital to recipient center distance, km	83 (54–102)
Donor hospital to recipient center travel time, min	64 (60–85)
**Intraoperative and perfusion variables**	
CPB time, min	178 (148–193)
VIS at OR exit	30 (30–40)
Inhaled nitric oxide use, *n* (%)	17 (100)
ECMO at OR exit, *n* (%)	0
Ventricular fibrillation at CPB weaning, *n* (%)	8 (47.1)
Temporary pacemaker use, *n* (%)	11 (64.7)
Asystole at end of surgery, *n* (%)	0
**Recipient characteristics and postoperative outcomes**	
Mechanical ventilation, h	16 (13–20)
Postoperative tracheostomy, *n* (%)	1 (5.9)
ICU stay, days	7 (5–11)
Hospital stay, days	34.5 (23–54)
Primary graft dysfunction (PGD), *n* (%)	0
ISHLT grade ≥ 2R at first biopsy, *n* (%)	0
Postoperative ECMO, *n* (%)	0
30‐day mortality, *n* (%)	0
Postoperative CRRT, *n* (%)	7 (41.2)

*Note:* Data are presented as median (interquartile range [IQR]) or *n* (%), as indicated.

Abbreviations: AWIT, asystolic warm ischemic time; CPB, cardiopulmonary bypass; CRRT, continuous renal replacement therapy; ECMO, extracorporeal membrane oxygenation; FWIT, functional warm ischemic time; ICU, intensive care unit; ISHLT, International Society for Heart and Lung Transplantation; OR, operating room; PGD, primary graft dysfunction; TA‐NRP, thoracoabdominal normothermic regional perfusion; VIS, vasoactive‐inotropic score; WLST, withdrawal of life‐sustaining therapy.

**FIGURE 2:**
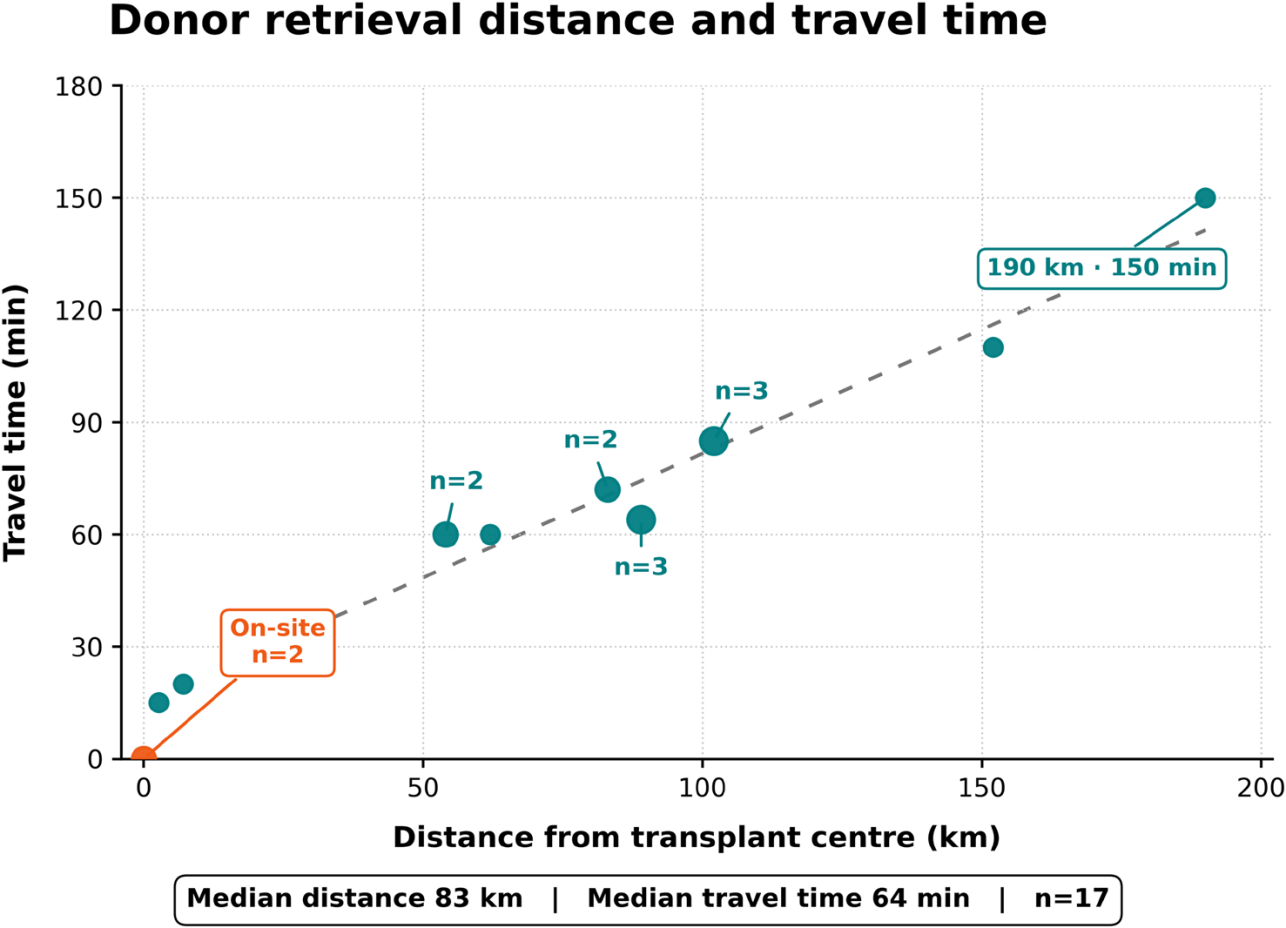
Donor retrieval distance and travel time. Bubble size represents the number of retrievals sharing the same distance–time combination. Retrievals performed at the recipient's transplant center are shown at the origin. The dashed line represents the linear trend for interhospital transfers. Overall, 17 retrievals were included.

### 3.4 Intraoperative and Postoperative Course

Intraoperative and early postoperative outcomes are reported in Table 3. Median cardiopulmonary bypass (CPB) time was 178 min (IQR, 148–193). The median vasoactive‐inotropic score (VIS) at operating room exit was 30 (IQR, 30–40). Inhaled nitric oxide (iNO) was administered in all cases. No recipient required ECMO support after leaving the operating room (0%), and temporary pacemaker (PM) support was required in 64.7% of cases. Ventricular fibrillation during weaning from the CPB occurred in 47.1% of cases, whereas asystole at the end of the surgery was not observed. Overall, the intraoperative course was stable in most patients, with consistent achievement of successful weaning from cardiopulmonary bypass and no requirement for postoperative mechanical circulatory support.

Recipients were mechanically ventilated for a median of 16 h (IQR, 13–20). Postoperative tracheostomy was required in 5.9% of recipients. The median ICU stay was 7 days (IQR, 5–11), whereas the median hospital stay was 34.5 days (IQR, 23–54). No recipient developed primary PGD, moderate‐to‐severe ACR (ISHLT grade ≥ 2R) at first biopsy, required postoperative VA‐ECMO, or died within 30 days after transplantation. Continuous renal replacement therapy was required in 41.2% of evaluable patients during the postoperative period.

## 4 Discussion

This case series describes the experience with cDCD heart procurement using TA‐NRP in hospitals without cardiac surgery onsite within a regional Italian program. The successful transplantation rate of 89.5% observed in this study is consistent with outcomes reported by larger programs worldwide, despite the mandatory 20‐min no‐touch period required by Italian law and the related concern regarding the impact of prolonged WIT on graft viability. The novelty of the present study lies in the combination of three elements not previously described together in a single report [16]: (1) the largest Italian cardiac DCD cohort reported to date; (2) a fully described mobile cardiac team performing TA‐NRP via sternotomy in hospitals without onsite cardiac surgery; and (3) excellent early recipient outcomes despite universally prolonged FWIT imposed by Italy's legal framework. Finally, this report highlights the role of anesthesiologists throughout the donation and procurement pathway, an aspect that remains underexplored in the current literature.

### 4.1 Relationship to Previous Studies

The principal published international experiences with cDCD heart transplantation using TA‐NRP are summarized in Table 4. Our findings question the universal applicability of recently proposed warm ischemia threshold derived from recent US data. In a cohort of 143 NRP‐recovered DCD heart transplants, identified an AWIT ≥ 10 min as an independent predictor of both 90‐day mortality (HR 6.42) and severe PGD (OR 2.99). In contrast, the median AWIT time in our series was 33 (IQR, 29.5–40) minutes, substantially exceeding this threshold, yet no recipient experienced severe PGD or died within 30 days. Similarly, [22] reported reduced 1‐year survival among DPP recipients exposed to FWIT ≥ 30 min, whereas all transplanted hearts in our cohort exceeded this threshold. A UK program, using a 5‐min stand‐off period in 188 DCD heart transplants, reported a survival outcome comparable to DBD [23]. Notably, the addition of the abdominal NRP to DPP failed to improve outcome despite prolonging the ischemic interval [22, 23]. These apparent discrepancies should be interpreted cautiously because ischemic time alone may not fully capture the biological context in which ischemic injury develops. The Italian TA‐NRP model differs fundamentally from both DPP‐based programs and NRP protocols implemented after substantially shorter stand‐off periods. Unlike DPP, TA‐NRP is used as the primary procurement strategy and is specifically intended to recondition the graft following the mandatory 20‐min no‐touch period imposed by Italian legislation. Importantly, several international experiences have already demonstrated that FWIT values exceeding 30 min [24] may remain compatible with favorable short‐ and long‐term outcomes [25, 26]. From this perspective, the median FWIT of 41 min (IQR, 37.5–52) observed in our cohort should not be viewed as an isolated outlier but rather as additional evidence consistent with published observations [27].

**TABLE 4:** Principal international experiences with cDCD heart transplantation using TA‐NRP.

Study	Country	Procurement and preservation strategy	Stand‐off period	TA‐NRP heart transplants, *n*	Reported FWIT	Early survival	PGD
Miñambres et al., 2021 [17]	Spain	TA‐NRP followed by static cold storage	5 min	4	8–16 min	100% alive and discharged	Not specifically reported; no postoperative MCS
Vandendriessche et al., 2021 [18]	Belgium	TA‐NRP followed by static cold storage	5 min	4	WLST‐to‐NRP: median 22 min (range 18–31)	30‐day survival 100%	Mild PGD: 1/4 (25%); no severe PGD or postoperative MCS
Louca et al., 2023 [19]	UK, Spain, US, Belgium	TA‐NRP followed by static cold storage or ex situ machine perfusion, according to center	Center‐specific; not reported in aggregate	157	Mean FWIT‐to‐reperfusion interval: 16.7 min	30‐day survival 96.8%; 1‐year survival 93.2%	Severe PGD not reported separately; postoperative ECMO 5.7%
Benkert et al., 2024 [20]	US	NRP followed predominantly by static cold storage	Not reported in registry analysis	296	Not reported	30‐day survival 98.6%	Not reported
Louca et al., 2025 [21]	Belgium, Spain, UK, and US	TA‐NRP compared with direct procurement and perfusion	Center‐specific	223	Median 15 min	1‐year survival 91%	Severe PGD 7.6%
Present study, 2026	Italy	Mobile‐team TA‐NRP followed by static cold storage	20 min	17	41 (37.5–52) min	30‐day survival 100%	0%

*Note:* Warm ischemic intervals were defined differently across studies and should not be considered directly equivalent. Miñambres et al. defined FWIT as the interval from systolic arterial pressure < 60 mmHg to TA‐NRP initiation. Louca et al. reported the interval from the onset of functional warm ischemia to reperfusion. Vandendriessche et al. reported WLST‐to‐NRP time, which should not be interpreted as an identically defined FWIT. In Benkert et al., the procurement strategy was inferred from registry variables because UNOS did not directly record normothermic regional perfusion versus direct procurement and perfusion during the study period; these cases are therefore described as registry‐classified NRP. The multicenter and registry cohorts may overlap with patients included in earlier national or single‐center reports. Absence of postoperative MCS should not be interpreted as absence of PGD unless PGD was formally adjudicated according to ISHLT criteria.

Abbreviations: MCS, mechanical circulatory support; NRP, normothermic regional perfusion; PGD, primary graft dysfunction; TA‐NRP, thoracoabdominal normothermic regional perfusion; UK, United Kingdom; UNOS, United Network for Organ Sharing; US, United States; WLST, withdrawal of life‐sustaining treatment.

Several factors may explain why our results differ from previously proposed ischemic thresholds: (1) TA‐NRP provides in situ reconditioning and metabolic recovery, which may “rescue” hearts from warm ischemic injury in a manner not achievable with direct procurement and perfusion (DPP); (2) the Italian program may employ more stringent donor selection criteria; and (3) the US thresholds derived from cohorts exposed to shorter stand‐off periods may reflect donor instability during the agonal phase rather than the consequences of a fixed, legally mandated asystolic interval. In Italy, the 20‐min no‐touch period occurs after complete circulatory arrest, when myocardial oxygen consumption is markedly reduced and the physiological conditions differ deeply from those observed during ongoing agonal deterioration. Additional evidence supports this interpretation. Wang et al. reported that FWIT > 23 min was associated with increased PGD rates but not with mortality, suggesting that prolonged warm ischemia alone should not necessarily justify graft discard [28]. Furthermore, Louca et al., in the largest multicenter comparison available, observed lower rates of PGD and rejection with TA‐NRP than with DPP, supporting the concept that in situ reperfusion may confer a biological advantage and potentially expand the tolerable warm ischemia window [19]. Mechanistically, TA‐NRP restores coronary perfusion under near‐physiologic conditions, allowing correction of metabolic acidosis, recovery of organized myocardial contraction, and real‐time comprehensive functional reassessment before final graft acceptance. In our program, reperfusion followed a standardized flow‐controlled protocol that combined early left‐ventricular unloading with gradual biventricular reloading as myocardial contractility recovered. We did not use a predefined pressure‐controlled reperfusion ramp or a dedicated pharmacological post‐conditioning protocol; vasoactive and inotropic support was individualized according to hemodynamic, echocardiographic and metabolic findings. Together with systematic metabolic optimization and serial TEE assessment, these protocol features may have supported functional recovery, although the present study was not designed to determine their independent cardioprotective effects. The biological plausibility of this strategy is supported by preclinical evidence. In an isolated rat DCD heart model, Sanz et al. [29] demonstrated that targeted reperfusion strategies—mild hypothermia, mechanical post‐conditioning, hypoxia—improved oxygen‐consumption‐cardiac‐work coupling, increased tissue ATP content, and favorably modulated mitochondrial function through complex I activity stimulation, reduced cytochrome c release, and upregulation of PGC‐1α (a regulator of mitochondrial biogenesis). Additional experimental data by Iyer et al. [30] further suggest that optimized reperfusion may enhance myocardial tolerance to warm ischemia through pharmacologic and mitochondrial protective mechanisms.

### 4.2 Significance of the Study Findings

The present experience has practical implications for the organization of cDCD heart procurement programs. A regional mobile team capable of performing complex cardiac procurement procedures in peripheral hospitals may help extend access to heart donation beyond centers with onsite cardiac surgery. This model represents a pragmatic extension of centralized expertise across a regional network, reducing inequity due to geographic area and the dependence of heart donation feasibility on donor hospital location. A further relevant aspect is the central role of multidisciplinary coordination throughout the entire pathway. In particular, the anesthesiologist plays a key role linking intensive care, extracorporeal circulation, cardiac surgery, and the transplantation team. This role extends beyond ensuring high‐quality palliative sedation during WLST and includes continuous monitoring during the dying process, documentation of the circulatory arrest, management of reperfusion physiology, and serial TEE assessment during TA‐NRP [13, 30, 31]. TEE is particularly valuable during graft evaluation. Serial evaluations allow interpretation of dynamic changes in biventricular function, wall motion, septal behavior, and valvular competence, transforming a warm‐ischemic donor heart into an organ that could be physiologically evaluated, optimized, and accepted with greater confidence [32]. Our experience therefore supports previous observations emphasizing the need for structured protocols, multidisciplinary training, and team familiarity with the physiologic and logistical challenges related to TA‐NRP.

Overall, the Bologna experience confirms that donor hearts exposed to prolonged FWIT can be successfully transplanted, and that this strategy can be implemented in hospitals without onsite cardiac surgery through a structured regional mobile heart team. From this standpoint, our experience serves as a bridge between Italian organizational innovation and the growing international literature on cardiac DCD transplantation, suggesting that warm ischemia thresholds derived from programs with shorter stand‐off periods may not be directly applicable when TA‐NRP reconditioning is employed.

### 4.3 Strengths and Limitations

Our findings should be interpreted in light of several strengths. This study provides a real‐world experience of successful DCD heart procurement with TA‐NRP in hospitals without onsite cardiac surgery, despite the restrictive Italian legal framework requiring a mandatory 20‐min no‐touch period. It also describes a reproducible mobile heart team workflow and provides detailed procedural timing, reperfusion, and early recipient outcome data that may assist other programs operating under similar logistical or regulatory constraints. Several limitations should also be acknowledged. This was a retrospective single‐center case series with a small sample size and no comparator group. The experience reflects a single regional program and may not be generalizable to other settings with different regulatory, logistical, or organizational conditions. However, these limitations are intrinsic to early cDCD program reporting, and the study provides valuable data from a context with unique legislative constraints. This study focuses on procedural feasibility, graft utilization, and early postoperative outcomes. Follow‐up was heterogeneous because the cohort included recently transplanted recipients. In addition, survival, rejection, graft function, and echocardiographic data beyond 30 days were not collected at uniform time points. Longer‐term graft durability and recipient outcomes therefore cannot be inferred from this analysis. Prospective follow‐up of the cohort will be required to address these outcomes.

## 5 Conclusion

Our experience suggests that cDCD heart procurement with TA‐NRP may be feasible in hospitals without onsite cardiac surgery when supported by a regional mobile‐team program. Despite the constraints imposed by the Italian legal framework, early posttransplant outcomes were favorable among carefully selected donors and recipients. Given the descriptive nature of the study, small sample size, absence of a comparator group, and limited follow‐up, these findings should be considered hypothesis‐generating and do not establish that current FWIT thresholds should be modified. Larger comparative studies with longer follow‐up are required to confirm these observations.

## Author Contributions

**Maria Benedetto:** conceptualization, investigation, methodology, data curation, writing – original draft. **Valentina Roncarati:** conceptualization, supervision, study design, investigation, methodology. **Arianna Campana:** supervision, study design, methodology. **Erika Cordella:** data curation, data analysis. **Domenico Nocera:** review and editing. **Sofia Martin‐Suarez:** data curation, review and editing. **Luca Botta:** review and editing. **Davide Pacini:** supervision, study design. **Letizia Perinati:** methodology, investigation, writing – original draft, review and editing. **Massimo Baiocchi:** review and editing. **Tommaso Tonetti:** methodology, investigation, writing – review and editing. **Fabrizio Monaco:** supervision, study design, methodology, data analysis, writing – review and editing.

## Conflicts of Interest

The authors declare no conflicts of interest.

## Supporting information


**Table S1A:** Case‐level donor characteristics.
**Table S1B:** Case‐level recipient characteristics.
**Table S1C:** Case‐level donor–recipient matching characteristics.
**Table S2A:** Case‐level procedural and ischemic characteristics.
**Table S2B:** Case‐level recipient and early postoperative outcomes.
**Table S2C:** Case‐level intraoperative and perfusion variables.

## Data Availability

The data that support the findings of this study are available on request from the corresponding author. The data are not publicly available due to privacy or ethical restrictions.

## References

[1] K. K. Dhital , A. Iyer , M. Connellan , et al., “Adult Heart Transplantation With Distant Procurement and Ex‐Vivo Preservation of Donor Hearts After Circulatory Death: A Case Series,” Lancet 385 (2015): 2585–2591. 10.1016/S0140-6736(15)60038-1 25888085

[2] S. Messer , A. Page , R. Axell , et al., “Outcome After Heart Transplantation From Donation After Circulatory‐Determined Death Donors,” Journal of Heart and Lung Transplantation 36 (2017): 1311–1318. 10.1016/j.healun.2017.10.021 29173394

[3] H. K. Siddiqi , J. Trahanas , M. Xu , et al., “Outcomes of Heart Transplant Donation After Circulatory Death,” Journal of the American College of Cardiology 82 (2023): 1512–1520. 10.1016/j.jacc.2023.08.006 37793748

[4] J. H. Kwon , A. D. Ghannam , K. Shorbaji , et al., “Early Outcomes of Heart Transplantation Using Donation After Circulatory Death Donors in the United States,” Circulation Heart Failure 15 (2022): e009844. 10.1161/CIRCHEARTFAILURE.122.009844 36214116

[5] P. Frati , V. Fineschi , M. Gulino , G. Montanari Vergallo , N. M. Di Luca , and E. Turillazzi , “Ethical and Legal Implications of Elective Ventilation and Organ Transplantation: ‘Medicalization’ of Dying Versus Medical Mission,” BioMed Research International 2014 (2014): 973758. 10.1155/2014/973758 PMC4122128 25126582

[6] A. N. Costa , L. Lombardini , D. Storani , and CNT Working Group , “Organ Procurement and Transplantation in Italy,” Transplantation 103, no. 6 (2019): 1065–1069. 10.1097/TP.0000000000002570 31246927

[7] M. Vergano , E. Magavern , F. Baroncelli , et al., “Making a Case for Controlled Organ Donation After Cardiac Death: The Story of Italy's First Experience,” Journal of Critical Care 38 (2017): 129–131. 10.1016/j.jcrc.2016.10.028 27888715

[8] S. D. Shemie , A. J. Baker , G. Knoll , et al., “International Guideline Development for the Determination of Death,” Intensive Care Medicine 40, no. 6 (2014): 788–797. 10.1007/s00134-014-3242-7 PMC4028548 24664151

[9] S. D. Shemie , “National Recommendations for Donation After Cardiocirculatory Death in Canada: Donation After Cardiocirculatory Death in Canada,” CMAJ: Canadian Medical Association Journal 175, no. 8 (2006): S1. 10.1503/cmaj.060895 PMC1635157 17124739

[10] I. Ortega‐Deballon , L. Hornby , and S. D. Shemie , “Protocols for Uncontrolled Donation After Circulatory Death: A Systematic Review of International Guidelines, Practices and Transplant Outcomes,” Critical Care 19 (2015): 268. 10.1186/s13054-015-0985-7 PMC4495857 26104293

[11] G. C. Oniscu , L. V. Randle , P. Muiesan , et al., “In Situ Normothermic Regional Perfusion for Controlled Donation After Circulatory Death—The United Kingdom Experience,” American Journal of Transplantation 14 (2014): 2846–2854. 10.1111/ajt.12927 25283987

[12] C. Lazzeri , M. Bonizzoli , G. Feltrin , and A. Peris , “Normothermic Regional Perfusion Mobile Teams in Controlled Donation After Circulatory Death Pathway: Evidence and Peculiarities,” World Journal of Transplantation 14 (2024): 97860. 10.5500/wjt.v14.i4.97860 PMC11438942 39697456

[13] L. Orsi and G. R. Gristina , “Palliative Sedation: The Position Statement of the Italian National Committee for Bioethics,” Minerva Anestesiologica 83 (2017): 524–528, 10.23736/S0375-9393.16.11736-5. 28006887

[14] J. Kobashigawa , A. Zuckermann , P. Macdonald , et al., “Report From a Consensus Conference on Primary Graft Dysfunction After Cardiac Transplantation,” Journal of Heart and Lung Transplantation 33 (2014): 327–340, 10.1016/j.healun.2014.02.027. 24661451

[15] M. V. Antonini , S. Martin‐Suàrez , L. Botta , et al., “Thoracoabdominal Normothermic Regional Perfusion Using Mobile Closed Extracorporeal Circuit in Circulatory Death Determination Heart Donors,” ASAIO Journal 71 (2025): e61–e65, 10.1097/MAT.0000000000002282. 39145661

[16] S. Martin‐Suarez , L. Botta , G. Barberio , et al., “Cardiac Function Recovery After 20‐Min Hands‐Off Using Thoracoabdominal‐Normothermic Regional Perfusion,” Journal of Heart and Lung Transplantation 45 (2026): 324–333, 10.1016/j.healun.2025.09.003. 40947079

[17] E. Miñambres , M. Royo‐Villanova , M. Pérez‐Redondo , et al., “Spanish Experience With Heart Transplants From Controlled Donation After the Circulatory Determination of Death Using Thoraco‐Abdominal Normothermic Regional Perfusion and Cold Storage,” American Journal of Transplantation 21 (2021): 1597–1602, 10.1111/ajt.16446. 33319435

[18] K. Vandendriessche , V. Tchana‐Sato , D. Ledoux , et al., “Transplantation of Donor Hearts After Circulatory Death Using Normothermic Regional Perfusion and Cold Storage Preservation,” European Journal of Cardio‐Thoracic Surgery 60 (2021): 813–819, 10.1093/ejcts/ezab139. 33783513

[19] J. Louca , M. Öchsner , A. Shah , et al., “The International Experience of In‐Situ Recovery of the DCD Heart: A Multicentre Retrospective Observational Study,” EClinicalMedicine 58 (2023): 101887. 10.1016/j.eclinm.2023.101887 PMC9995283 36911270

[20] A. R. Benkert , J. E. Keenan , J. N. Schroder , et al., “Early U.S. Heart Transplant Experience With Normothermic Regional Perfusion Following Donation After Circulatory Death,” JACC. Heart Failure 12 (2024): 2073–2083, 10.1016/j.jchf.2024.06.007. 39093259

[21] J. O. Louca , M. Öchsner , S. Bhagra , et al., “Outcomes After Donation After Circulatory Determination of Death Cardiac Transplantation: An International, Multicenter Retrospective Study,” JACC. Heart Failure 13 (2025): 102473, 10.1016/j.jchf.2025.04.003. 40293864

[22] Y. Hong , U. Nasim , N. Iyanna , et al., “Effects of Functional Warm Ischemia Time on Posttransplant Outcomes in Donation After Circulatory Death Heart Transplantation Using Direct Procurement and Perfusion,” Transplantation 110 (2026): e1284–e1293, 10.1097/TP.0000000000005725. 42044284

[23] K. Morcos , L. Simmonds , S. Rushton , et al., “The United Kingdom National Programme of DCD Heart Transplantation: Timings, Techniques, Feasibility, and Outcomes With Abdominal Normothermic Regional Perfusion,” Journal of Heart and Lung Transplantation 45, no. 10 (2026): 1581–1598, 10.1016/j.healun.2026.04.027. 42092411

[24] M. L. Goodwin , I. C. Nickel , H. Li , et al., “Direct Procurement With Machine Perfusion and Normothermic Regional Perfusion in Donation After Circulatory Death Heart Transplantation,” Journal of Thoracic and Cardiovascular Surgery 170 (2025): 256–265.e6. 10.1016/j.jtcvs.2024.10.033 39490962

[25] A. Brann , B. Jackson , R. White , et al., “(191) Impact of Functional Warm Ischemic Time on Short Term Outcomes in Donation After Circulatory Death Heart Transplantation,” Journal of Heart and Lung Transplantation 42 (2023): S94.

[26] C. Pasrija , A. DeBose‐Scarlett , C. Keck , et al., “(192) Prolonged Warm Ischemic Time Is Safe for Cardiac Donation After Circulatory Death,” Journal of Heart and Lung Transplantation 42 (2023): S94–S95.

[27] S. E. Scheuer , P. C. Jansz , and P. S. Macdonald , “Heart Transplantation Following Donation After Circulatory Death: Expanding the Donor Pool,” Journal of Heart and Lung Transplantation 40 (2021): 882–889. 10.1016/j.healun.2021.03.011 33994229

[28] C. C. Wang , M. Petrovic , A. Ahmad , et al., “The Effect of Warm Ischemic Intervals on Primary Graft Dysfunction in Normothermic Regional Perfusion for Donation After Circulatory Death Heart Transplant,” J Thorac Cardiovasc Surg 170, no. 4 (2025): 1109–1116.e3, 10.1016/j.jtcvs.2025.03.037. 40319402

[29] M. N. Sanz , E. Farine , P. Niederberger , et al., “Cardioprotective Reperfusion Strategies Differentially Affect Mitochondria: Studies in an Isolated Rat Heart Model of Donation After Circulatory Death (DCD),” American Journal of Transplantation 19 (2019): 331–344, 10.1111/ajt.15024. 30019521

[30] A. Iyer , L. Gao , A. Doyle , et al., “Increasing the Tolerance of DCD Hearts to Warm Ischemia by Pharmacological Postconditioning,” American Journal of Transplantation 14 (2014): 1744–1752. 10.1111/ajt.12782 25040306

[31] M. Zanierato and A. Rubino , “The Anesthetist Perspective: Optimization of Cardiac Allograft From Withdrawal of Life Support to Reperfusion in the Controlled Donation After Circulatory Death,” Annals of Cardiothoracic Surgery 14 (2025): 58–60. 10.21037/acs-2024-dcd-0098 PMC11811568 39944499

[32] C. Vandenbriele , J. Brouckaert , G. Hans , et al., “The Role of Transesophageal Echocardiography in Guiding Heart Donation After Circulatory Death,” Clinical Transplantation 36 (2022): e14783, 10.1111/ctr.14783. 35920407

