# Supplementary material for: Heart Donation After Controlled Circulatory Death in Noncardiac Surgery Hospitals With a Mobile Heart Team: A Case Series From an Italian Regional Program and Integrated Review of the Literature
Source: Artif Organs. 2026 Sep 27;50(11):1657–67. doi: 10.1111/aor.70255 (PMC13720652; doi:10.1111/aor.70255)
Supplement: Supplementary file 1 [file AOR-50-1657-s001.docx]

**Supplemental Material**

**Heart Donation After Controlled Circulatory Death in Non-Cardiac Surgery Hospitals with a Mobile Heart Team. A Case Series from an Italian Regional Program and Integrated Review of the Literature**

Maria Benedetto, MD, Valentina Roncarati, MD, Arianna Campana, MD, Erika Cordella, MD, Domenico Nocera, MD, Sophia M Suarez, MD, Luca Botta, MD, Pacini Davide, MD, Letizia Perinati, MD, Massimo Baiocchi, MD, Tommaso Tonetti, MD, Fabrizio Monaco MD.

**************

**Table S1A.** Case-level donor characteristics. Page 2

**Table S1B.** Case-level recipient characteristics. Page 3

**Table S1C.** Case-level donor-recipient matching characteristics. Page 4

**Table S2A.** Case-level procedural and ischemic characteristics. Page 5

**Table S2B.** Case-level recipient and early postoperative outcomes. Page 6

**Table S2C.** Case-level intraoperative and perfusion variables. Page 7

**Table S1A. Case-level donor characteristics.**

| **Case** | **Age, y** | **Sex** | **Weight, kg** | **Height, cm** | **Cause of death** | **Hypertension** | **ABO group** | **Rh system** | **ICU stay before procurement, d** |
| --- | --- | --- | --- | --- | --- | --- | --- | --- | --- |
| DCD-01 | 23 | M | 80 | 180 | Post-anoxic encephalopathy | No | 0 | + | 12 |
| DCD-02 | 58 | M | 100 | 175 | Post-anoxic encephalopathy | Yes | 0 | + | 7 |
| DCD-03 | 46 | M | 70 | 180 | Post-anoxic encephalopathy | No | A | + | 10 |
| DCD-04 | 59 | M | 90 | 185 | Cerebral hemorrhage | No | A | − | 8 |
| DCD-05 | 49 | M | 76 | 170 | Post-anoxic encephalopathy | No | A | + | 5 |
| DCD-06 | 64 | F | 96 | 165 | Cerebral hemorrhage | No | 0 | + | 9 |
| DCD-07 | 54 | M | 82 | 175 | Post-anoxic encephalopathy | No | A | + | 12 |
| DCD-08 | 58 | M | 95 | 190 | Post-anoxic encephalopathy | No | A | − | 17 |
| DCD-09 | 51 | M | 82 | 185 | Cerebral hemorrhage | No | 0 | + | 8 |
| DCD-10 | 59 | M | 86 | 182 | Cerebral hemorrhage | Yes | 0 | − | 45 |
| DCD-11 | 55 | M | 100 | 176 | Cerebral hemorrhage | No | 0 | + | 34 |
| DCD-12 | 63 | M | 93 | 173 | Cerebral hemorrhage | No | A | + | 21 |
| DCD-13 | 54 | F | 84 | 160 | Cerebral hemorrhage | Yes | A | + | 13 |
| DCD-14 | 44 | M | 70 | 175 | Post-anoxic encephalopathy | No | 0 | + | 11 |
| DCD-15 | 60 | F | 87 | 165 | Cerebral hemorrhage | Yes | A | + | 18 |
| DCD-16 | 38 | M | 75 | 178 | Post-anoxic encephalopathy | No | B | + | 7 |
| DCD-17 | 57 | M | 65 | 175 | Post-anoxic encephalopathy | No | AB | − | 13 |
| DCD-18 | 64 | M | 73 | 180 | Post-anoxic encephalopathy | No | A | + | 8 |
| DCD-19 | 68 | F | 70 | 165 | Cerebral hemorrhage | No | 0 | − | 10 |

Abbreviations: DCD, donor after circulatory death; M, male; F, female; ABO, ABO blood group system; Rh, Rhesus blood group system; ICU, intensive care unit; d, days

**Table S1B. Case-level recipient characteristics.**

| **Case** | **Age, y** | **Sex** | **Weight,**  **Kg** | **Height,**  **cm** | **ABO group** | **Rh system** | **Indication for transplantation** | **Macro-area urgent status** | **Clinical setting at transplantation** | **Preoperative MCS** |
| --- | --- | --- | --- | --- | --- | --- | --- | --- | --- | --- |
| DCD-01 | 60 | M | 73 | 174 | 0 | + | Other cardiovascular disease | No | Outpatient | No |
| DCD-02 | 66 | M | 106 | 187 | 0 | + | Coronary artery disease | No | Outpatient | No |
| DCD-03 | 57 | M | 73 | 180 | A | + | Coronary artery disease | No | Outpatient | No |
| DCD-04 | 58 | M | 85 | 180 | A | - | Valvular heart disease | No | Outpatient | No |
| DCD-05 | 55 | M | 71 | 178 | A | + | Coronary artery disease | No | Outpatient | No |
| DCD-06 | 57 | F | 88 | 175 | 0 | + | Cardiomyopathy | No | Outpatient | No |
| DCD-07 | 59 | M | 71 | 175 | A | + | Coronary artery disease | No | Outpatient | No |
| DCD-08 | NA | NA | NA | NA | NA | NA | NA | NA | NA | NA |
| DCD-09 | 55 | M | 81 | 178 | 0 | + | Cardiomyopathy | No | Outpatient | No |
| DCD-10 | 58 | M | 95 | 170 | 0 | - | Cardiomyopathy | No | Regular ward | No |
| DCD-11 | 46 | M | 91 | 178 | 0 | + | Cardiomyopathy | No | Outpatient | No |
| DCD-12 | 57 | M | 90 | 173 | A | + | Cardiomyopathy | No | Outpatient | No |
| DCD-13 | NA | NA | NA | NA | NA | NA | NA | NA | NA | NA |
| DCD-14 | 57 | M | 74 | 171 | 0 | + | Coronary artery disease | No | Regular ward | No |
| DCD-15 | 48 | F | 60 | 157 | A | + | Valvular heart disease | No | Outpatient | No |
| DCD-16 | 61 | F | 58 | 182 | B | + | Coronary artery disease | No | Outpatient | No |
| DCD-17 | 39 | F | 59 | 167 | AB | + | Congenital heart disease | No | Outpatient | No |
| DCD-18 | 62 | M | 72 | 183 | A | + | Cardiomyopathy | Yes | ICU | No |
| DCD-19 | 54 | F | 56 | 174 | 0 | + | Cardiomyopathy | No | Outpatient | No |

Cases DCD-08 and DCD-13 did not proceed to transplantation and therefore have no recipient-level data.

Abbreviations: DCD, Donor after circulatory death; M, male; F, female; NA, not available; MCS, mechanical circulatory support

**Table S1C. Case-level donor-recipient matching characteristics.**

| **Case** | **Sex pairing** | **Absolute age difference, y** | **Donor older than recipient** | **ABO matching** | **Rh pairing (D/R)** | **D/R weight ratio** | **D/R pHM ratio** |
| --- | --- | --- | --- | --- | --- | --- | --- |
| DCD-01 | Matched | 37 | No | Identical | +/+ | 1.10 | 1.08 |
| DCD-02 | Matched | 8 | No | Identical | +/+ | 0.94 | 0.93 |
| DCD-03 | Matched | 11 | No | Identical | +/+ | 0.96 | 0.97 |
| DCD-04 | Matched | 1 | Yes | Identical | -/- | 1.06 | 1.05 |
| DCD-05 | Matched | 6 | No | Identical | +/+ | 1.07 | 1.02 |
| DCD-06 | Matched | 7 | Yes | Identical | +/+ | 1.09 | 1.02 |
| DCD-07 | Matched | 5 | No | Identical | +/+ | 1.15 | 1.09 |
| DCD-08 | NA | NA | NA | NA | NA | NA | NA |
| DCD-09 | Matched | 4 | No | Identical | +/+ | 1.01 | 1.03 |
| DCD-10 | Matched | 1 | Yes | Identical | -/- | 0.91 | 0.98 |
| DCD-11 | Matched | 9 | Yes | Identical | +/+ | 1.10 | 1.05 |
| DCD-12 | Matched | 6 | Yes | Identical | +/+ | 1.03 | 1.02 |
| DCD-13 | NA | NA | NA | NA | NA | NA | NA |
| DCD-14 | Matched | 13 | No | Identical | +/+ | 0.95 | 0.98 |
| DCD-15 | Matched | 12 | Yes | Identical | +/+ | 1.45 | 1.30 |
| DCD-16 | M donor-F recipient | 23 | No | Identical | +/+ | 1.29 | 1.42 |
| DCD-17 | M donor-F recipient | 18 | Yes | Identical | -/+ | 1.10 | 1.34 |
| DCD-18 | Matched | 2 | Yes | Identical | +/+ | 1.01 | 1.00 |
| DCD-19 | Matched | 14 | Yes | Identical | -/+ | 1.25 | 1.12 |

Cases DCD-08 and DCD-13 did not proceed to transplantation and therefore have no recipient-level data.

Abbreviations: ABO, ABO blood group system; D/R, donor/recipient; DCD, donation after circulatory death; F, female; M, male; NA, not applicable; NR, not reported; pHM, predicted heart mass; Rh, Rhesus D antigen; y, years.

**Table S2A. Case-level procedural and ischemic characteristics.**

| **Case** | **Heart transplanted** | **FWIT >30 min** | **FWIT, min** | **WLST to agonal, min** | **Agonal phase, min** | **AWIT, min** | **Arrest to incision, min** | **Cold ischemia, min** | **Donor VIS at separation from TA-NRP support** | **Distance, km** | **Travel time, min** |
| --- | --- | --- | --- | --- | --- | --- | --- | --- | --- | --- | --- |
| DCD-01 | Yes | Yes | 41 | 3 | 15 | 29 | 31 | 181 | 30 | 62 | 60 |
| DCD-02 | Yes | Yes | 37 | 2 | 10 | 31 | 28 | 221 | 70 | 102 | 85 |
| DCD-03 | Yes | Yes | 35 | 6 | 14 | 25 | 21 | 68 | 30 | 0 | 0 |
| DCD-04 | Yes | Yes | 40 | 4 | 15 | 38 | 25 | 203 | 70 | 83 | 72 |
| DCD-05 | Yes | Yes | 35 | 4 | 6 | 37 | 31 | 228 | 110 | 89 | 64 |
| DCD-06 | Yes | Yes | 66 | 1 | 23 | 48 | 22 | 170 | 0 | 89 | 64 |
| DCD-07 | Yes | Yes | 33 | 3 | 8 | 27 | 26 | 195 | 0 | 89 | 64 |
| DCD-08 | No | Yes | 44 | 1 | 8 | 42 | 39 | NA | 60 | NA | NA |
| DCD-09 | Yes | Yes | 59 | 2 | 23 | 45 | 28 | 200 | 10 | 83 | 72 |
| DCD-10 | Yes | Yes | 55 | 2 | 29 | 30 | 22 | 147 | 10 | 2.7 | 15 |
| DCD-11 | Yes | Yes | 52 | 3 | 16 | 50 | 33 | 189 | 80 | 102 | 85 |
| DCD-12 | Yes | Yes | 32 | 4 | 14 | 33 | 26 | 206 | 50 | 54 | 60 |
| DCD-13 | No | Yes | 41 | 2 | 14 | 33 | 25 | NA | 0 | NA | NA |
| DCD-14 | Yes | Yes | 38 | 1 | 6 | 36 | 22 | 180 | 30 | 190 | 150 |
| DCD-15 | Yes | Yes | 40 | 2 | 11 | 31 | 24 | 240 | 30 | 54 | 60 |
| DCD-16 | Yes | Yes | 40 | 1 | 11 | 32 | 24 | 120 | 40 | 0 | 0 |
| DCD-17 | Yes | Yes | 52 | 4 | 26 | 26 | 21 | 273 | 40 | 152 | 110 |
| DCD-18 | Yes | Yes | 52 | 3 | 6 | 46 | 45 | 146 | 30 | 102 | 85 |
| DCD-19 | Yes | Yes | 53 | 3 | 28 | 26 | 25 | 137 | 40 | 7.1 | 20 |
| Cases DCD-08 and DCD-13 did not proceed to transplantation and therefore have no recipient-level data.  Abbreviations: min, minute; km, kilometre; DCD, donor after circulatory death; FWIT, functional warm ischemic time; WLST, withdrawal of life-sustaining treatment, AWIT, asystolic warm ischemic time, TA-NRP, thoracoabdominal normothermic regional perfusion. | | | | | | | | | | | |

**Table S2B. Case-level recipient and early postoperative outcomes.**

| **Case** | **Mechanical ventilation, h** | **Tracheostomy** | **ICU stay, d** | **Hospital stay, d** | **Primary graft dysfunction** | **ISHLT grade ≥2R at first biopsy** | **Postoperative ECMO** | **30-day mortality** | **Postoperative CRRT** |
| --- | --- | --- | --- | --- | --- | --- | --- | --- | --- |
| DCD-01 | 9 | No | 5 | 26 | No | No | No | No | No |
| DCD-02 | 13 | No | 4 | 42 | No | No | No | No | No |
| DCD-03 | 16 | No | 5 | 20 | No | No | No | No | No |
| DCD-04 | 16 | No | 6 | 20 | No | No | No | No | No |
| DCD-05 | 16 | No | 4 | 40 | No | No | No | No | No |
| DCD-06 | 45 | No | 11 | 34 | No | No | No | No | No |
| DCD-07 | 20 | No | 11 | 35 | No | No | No | No | Yes |
| DCD-09 | 12 | No | 6 | 11 | No | No | No | No | Yes |
| DCD-10 | 15 | No | 11 | 90 | No | No | No | No | Yes |
| DCD-11 | 16 | No | 7 | 24 | No | No | No | No | No |
| DCD-12 | 15 | No | 7 | 95 | No | No | No | No | Yes |
| DCD-14 | 84 | No | 10 | 131 | No | No | No | No | No |
| DCD-15 | 56 | No | 7 | NA | No | No | No | No | Yes |
| DCD-16 | 526 | Yes | 96 | 122 | No | No | No | No | Yes |
| DCD-17 | 16 | No | 11 | 37 | No | No | No | No | Yes |
| DCD-18 | 12 | No | 6 | 29 | No | No | No | No | No |
| DCD-19 | 13 | No | 5 | 21 | No | No | No | No | No |
| Cases DCD-08 and DCD-13 did not proceed to transplantation and therefore have no recipient-level data.  Abbreviations: h, hour, d, day; DCD, Donor after circulatory death; NA, not available; ISHLT , International Society for Heart and Lung Transplantation; ECMO, Extracorporeal membrane oxygenation;CRRT, continuous renal replacement therapy. | | | | | | | | | |

**Table S2C. Case-level intraoperative and perfusion variables.**

| **Case** | **CPB time, min** | **VIS at OR exit** | **Inhaled nitric oxide** | **ECMO at OR exit** | **Temporary pacemaker** | **Asystole at end of surgery** | **Ventricular fibrillation at CPB weaning** |
| --- | --- | --- | --- | --- | --- | --- | --- |
| DCD-01 | 192 | 50 | Yes | No | Yes | No | Yes |
| DCD-02 | 178 | 10 | Yes | No | Yes | No | Yes |
| DCD-03 | 132 | 20 | Yes | No | Yes | No | Yes |
| DCD-04 | 167 | 20 | Yes | No | Yes | No | No |
| DCD-05 | 148 | 30 | Yes | No | Yes | No | Yes |
| DCD-06 | 130 | 40 | Yes | No | Yes | No | Yes |
| DCD-07 | 253 | 30 | Yes | No | No | No | No |
| DCD-09 | 148 | 30 | Yes | No | No | No | No |
| DCD-10 | 199 | 30 | Yes | No | Yes | No | No |
| DCD-11 | 157 | 30 | Yes | No | Yes | No | No |
| DCD-12 | 188 | 170 | Yes | No | No | No | Yes |
| DCD-14 | 158 | 50 | Yes | No | No | No | No |
| DCD-15 | 212 | 50 | Yes | No | Yes | No | No |
| DCD-16 | 193 | 40 | Yes | No | Yes | No | yes |
| DCD-17 | 227 | 30 | Yes | No | No | No | Yes |
| DCD-18 | 137 | 30 | Yes | No | Yes | No | No |
| DCD-19 | 187 | 30 | Yes | No | No | No | No |
| Cases DCD-08 and DCD-13 did not proceed to transplantation and therefore have no recipient-level data.  Abbreviations: min, minute; DCD, donor after circulatory death; CPB, cardiopulmonary bypass; VIS, vasoactive-inotropic score; OR, operating room; ECMO, extracorporeal membrane oxygenation. | | | | | | | |
